# Synthesis, characterization and anticancer effect of doxorubicin-loaded dual stimuli-responsive smart nanopolymers

**DOI:** 10.3762/bjnano.15.96

**Published:** 2024-09-26

**Authors:** Ömür Acet, Pavel Kirsanov, Burcu Önal Acet, Inessa Halets-Bui, Dzmitry Shcharbin, Şeyda Ceylan Cömert, Mehmet Odabaşı

**Affiliations:** 1 Vocational School of Health Science, Pharmacy Services Program, Tarsus University, Tarsus, Turkey; 2 Institute of Biophysics and Cell Engineering of the National Academy of Sciences of Belarus, Minsk, Belarus; 3 Faculty of Arts and Science, Chemistry Department, Aksaray University, Aksaray, Turkey

**Keywords:** cancer cell line HeLa, cytotoxicity, doxorubicin, drug delivery, smart nanopolymers, temperature- and pH-sensitive nanopolymer

## Abstract

Nanopolymers represent a significant group of delivery vehicles for hydrophobic drugs. In particular, dual stimuli-responsive smart polymer nanomaterials might be extremely useful for drug delivery and release. We analyzed the possibility to include the known antitumor drug doxorubicin (DOX), which has antimitotic and antiproliferative effects, in a nanopolymer complex. Thus, doxorubicin-loaded temperature- and pH-sensitive smart nanopolymers (DOX-SNPs) were produced. Characterizations of the synthesized nanostructures were carried out including zeta potential measurements, Fourier-transform infrared spectroscopy, and scanning electron microscopy. The loading capacity of the nanopolymers for DOX was investigated, and encapsulation and release studies were carried out. In a final step, the cytotoxicity of the DOX–nanopolymer complexes against the HeLa cancer cell line at different concentrations and incubation times was studied. The DOX release depended on temperature and pH value of the release medium, with the highest release at pH 6.0 and 41 °C. This effect was similar to that observed for the commercial liposomal formulation of doxorubicin Doxil. The obtained results demonstrated that smart nanopolymers can be efficiently used to create new types of doxorubicin-based drugs.

## Introduction

Almost one in six deaths worldwide is from cancer, and cancer caused approximately 10 million deaths in 2020. Today, nanotechnology is emerging as an effective way to enable rapid diagnosis and treatment of cancer diseases [1–3]. The chemotherapy drug doxorubicin (DOX) has been used in the present study. It is a known antitumor antibiotic of the anthracycline series, which has been approved as anticancer drug in 1974. It has antimitotic and antiproliferative effects. The mechanism of action is interaction with DNA, the formation of free radicals, and a direct effect on cell membranes with the suppression of nucleic acid synthesis. The most hazardous side effect of DOX is dilated cardiomyopathy, which causes congestive heart failure [4]. To prevent side effects of doxorubicin, liposomal formulations were approved, namely “Myocet liposomal” and “Doxil”. The non-pegylated liposomal doxorubicin Myocet liposomal was approved in the European Union and in Canada for the therapy of metastatic breast cancer in combination with cyclophosphamide [5–6]. The FDA approved Doxil [4]. It was found that liposome-encapsulated DOX is less cardiotoxic than free DOX. To date, several types of nanoparticles, such as liposomes, micelles, and metal-organic frameworks, have been studied to encapsulate DOX to obtain effective and non-toxic drugs [7–8].

Great attention has been paid to nanoparticles because of their specific properties, such as small size, high stability, low toxicity, modifiable hydrophilicity/hydrophobicity, and the possibility of surface functionalization for targeted localization. Polymeric nanoparticles are a versatile approach to drug delivery (DD) with the potential to circumvent barriers associated with negative impacts on physiological functions. They can effectively transport therapeutic agents to targeted cells or specific intracellular regions through passive targeting or ligand-based strategies [9–11]. The use of certain polymers could potentially enable sustained drug levels for controlled release and extended durations. While numerous biodegradable polymeric nanoparticles derived from proteins or polysaccharides have been studied for drug delivery and controlled drug release in the recent past, the emphasis of research has now turned towards synthetic polymers, resulting in significant advancements in this field [9].

Certain designs in nanostructures are extremely useful to combat diseases [12–13]. Polymeric platforms have attracted great interest in recent years [14–20]. “Stimulus-sensitive” polymers (smart polymers) exhibit conformational changes or phase transition behavior in response to external stimuli [21–22]. Different “smart” polymeric nanoparticle systems have been described in the literature, which might respond to both internal and external stimuli to release drugs. Remarkable developments have been made regarding in vitro and in vivo drug release with varying drug loading levels [23]. Such smart polymer nanoparticles have been suggested in the literature, and their effectiveness has been proven by our group, especially for the loading and enhanced release of naringenin [20,24], another anticancer drug, and ʟ-asparaginase, a therapeutic enzyme [22].

Studies indicate that the response of polymeric materials to two or more stimuli significantly enhances drug delivery compared to nanoplatforms that respond to a single stimulus [24–25]. The difference between body temperature and ambient temperature, along with variations in pH value between the medium and the intended compartment, make thermo- and pH-sensitive polymers a crucial subset of smart polymers in drug delivery systems (DDSs). These responsive platforms must maintain stability at physiological pH levels [21].

Poly(*N*-isopropylacrylamide) (PNIPAM) stands out as one of the most common and most extensively researched thermosensitive polymers [21,24]; it is widely utilized in controlled drug release experiments [26–28] as its lower critical solution temperature (LCST) of 32 °C is near the body temperature [21]. It has been reported that vinyl imidazole (VIm) is a pH-responsive material [29]. The deprotonation of Vim around the physiological pH value may be sufficiently convenient for DDSs, particularly for the design of pH-sensitive platforms [30]. The pH value of normal tissues and blood (pH 7.4) is normally higher than the pH value of tumor cell endosomes and lysosomes (pH 6.5–6.8). Platforms that are sensitive to two factors, such as pH and temperature, can be engineered to enhance targeting efficacy while minimizing systemic side effects [31–32].

Here, a strategy for the production and application of DOX-SNPs is proposed. FTIR, SEM, and zeta potential measurements were performed to characterize the SNPs. In addition to experiments regarding the DOX loading capacity of the SNPs at different concentrations, the effect of pH and temperature on the release of DOX was also investigated. Finally, the cytotoxicity of DOX-SNPs against the cancer cell line HeLa at different concentrations and incubation times was studied.

## Experimental

### Materials

Doxorubicin and all other chemicals were purchased from Sigma-Aldrich (St. Louis, MO, USA). The water utilized in the experiments was purified by a Barnstead (Dubuque, IA, USA) ROpure LP^®^ reverse osmosis unit.

### Cell culture

Experiments were conducted using the HeLa cancer cell line (CCL-2 ATCC). Cells were grown in full DMEM with stable glutamine, 4.5 g/L glucose (Life Technologies, Paisley, U.K.), 10% FBS (Life Technologies, Paisley, U.K.), 100 U/mL penicillin, and 0.1 mg/mL streptomycin (Millipore-Sigma, Burlington, MA, United States) at 37 °C in a humidified air atmosphere with 5% CO_2_.

### Preparation of DOX-SNPs

**Figure 1:**
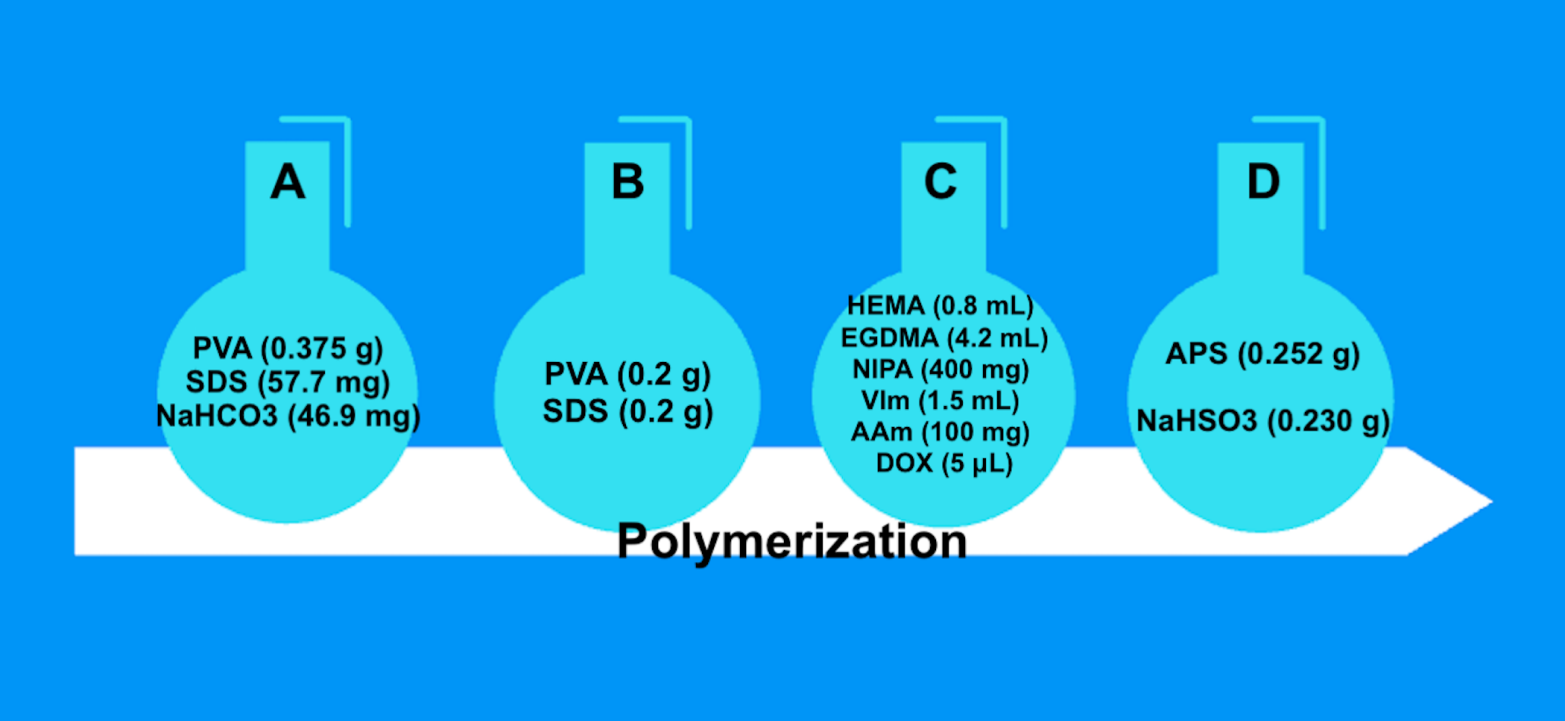
Solutions utilized during polymerization.

To synthesize DOX-containing nanoparticle structures, the miniemulsion polymerization method was used according to a protocol adapted from [24]. First, the water phase was formed. The water phase was obtained by adding and dissolving 0.375 g polyvinyl alcohol (PVA), 57.7 mg sodium dodecyl sulfate (SDS), and 46.9 mg NaHCO_3_ in 20 mL of water (solution A in Figure 1). For solution B, 0.2 g PVA and 0.2 g SDS were dissolved in 400 mL of water. The organic phase (solution C) was obtained by mixing and dissolving (30 min in a beaker) 0.8 mL 2-hydroxyethyl methacrylate (HEMA), 4.2 mL ethylene glycol dimethacrylate (EGDMA), 400 mg *N*-isopropylacrylamide (NIPA), 1.5 mL VIm, 100 mg acrylamide (AAm), and 5 µL of 5 µmol/L DOX solution. Subsequently, the first water phase was added to the organic phase and stirred for 10 min with the aid of a homogenizer (T25B, Ika Labortechnik, Germany). Then, the mixture was stirred with a mechanical stirrer in a two-neck flask until the temperature reached 50 °C. This mixture (solution A + solution C) was added to the solution B and mixed with a mechanical mixer (500 rpm). Last, 0.252 g ammonium persulfate (APS) and 0.230 g NaHSO_3_ (solution D) were added to the medium, and polymerization was initiated. After about 10 h of polymerization, the surfactants and unreacted monomers were washed out with the help of an ethanol–water mixture, and the mixture was centrifuged at 25,000 rpm (Beckman Coulter, Allegra 64R Centrifugen, USA). The precipitated nanostructures were redispersed in distilled water utilizing a sonicator and dried with a lyophilizer. Then, the nanostructures were stored at 4 °C.

### Characterization studies

Functional group analysis was carried out by Fourier-transform infrared (FTIR) spectroscopy. Scanning electron microscopy (SEM, Leo 440) was utilized for morphological characterizations of the SNPs; lyophilized SNPs were coated with gold, and the samples were placed in the SEM. The zeta potential the of DOX-SNPs was measured by using a Nano Zetasizer (NanoS, Malvern Instruments, London, UK).

### Cytotoxicity test

Cell viability was assessed using 3-(4,5-dimethylthiazol-2-yl)-2,5-diphenyltetrazolium bromide (MTT). The method is based on the ability of NADPH-dependent cellular oxidoreductase in living, metabolically active cells to reduce MTT to water-insoluble crystals of formazan. Cells were seeded onto clear 96-well plates at a concentration of 1 × 10^5^ cells/mL in a volume of 100 µL of cell suspension in fresh complete culture medium (DMEM with stable glutamine, 4.5 g/L glucose, 10% FBS, 100 U/mL penicillin, and 0.1 mg/mL streptomycin). The cells were preincubated for 24 h for cell adhesion and their transition to the active phase of reproduction. The nanomaterials were added to the cell suspension in the wells. For this, complexes of DOX-SNPs were prepared as described in section “Preparation of DOX-SNPs” to equivalents of loaded DOX of 1, 3, and 5 µmol/L in 0.05 mol/L phosphate-buffered saline (PBS), pH 7.4. The effect on the cancer cell line HeLa was studied after incubation for 24, 48, 72, and 96 h at 37 °C. After incubation, the medium was discarded from the wells, and 50 µL of MTT (0.5 mg/mL in 0.05 mol/L PBS, pH 7.4) were added to each well. The incubation time with MTT is 4 h. After the incubation, formazan crystals were dissolved in DMSO (100 µL added to each well). Then the optical absorption was measured on a microplate spectrophotometer Wallac Victor 2 (Perkin Elmer, USA) at wavelengths of 570 and 720 nm. The viability of cells (in relative units, r.u.) was calculated relatively to the control (non-treated cells).

### Statistical analysis

The statistical analysis of data was made by ANOVA with post-hoc Newman–Keuls test. The data are presented as mean ± S.D., *N* ≥ 6.

## Results and Discussion

### Characterization studies

**Figure 2:**
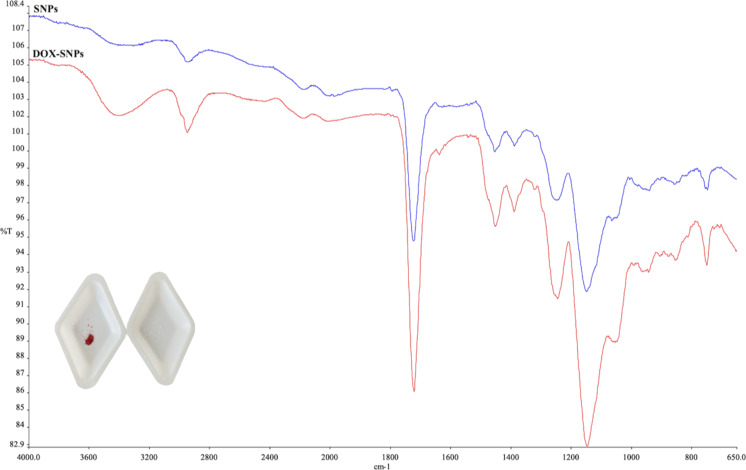
FTIR spectra of SNPs and DOX-SNPs.

The SNPs were synthesized as both thermo- and pH-sensitive nanostructures, and analyses of the characteristic peaks in the FTIR spectrum were performed. The FTIR spectra of SNPs and DOX-SNPs are given in Figure 2. From these spectra, the intensity of The FTIR spectra of SNPs and DOX-SNPs are given in Figure 2. The intensity of the OH peaks around 3370 cm^−1^ increased with the inclusion of the active molecule DOX in the structure. Also, the peak around 1722 cm^−1^, attributed to C=O stretching bands in SNPs, shifted with increasing intensity to 1700 cm^−1^ after addition of DOX to polymeric structure. N–H scissoring and NH bending bands around 1620 cm^−1^ showed up with increasing intensity in DOX-SNPs [33]. The inset in Figure 2 shows DOX-SNPs (red) and pure SNPs (white). Based on these results, the successful incorporation of DOX into SNPs has been demonstrated.

**Figure 3:**
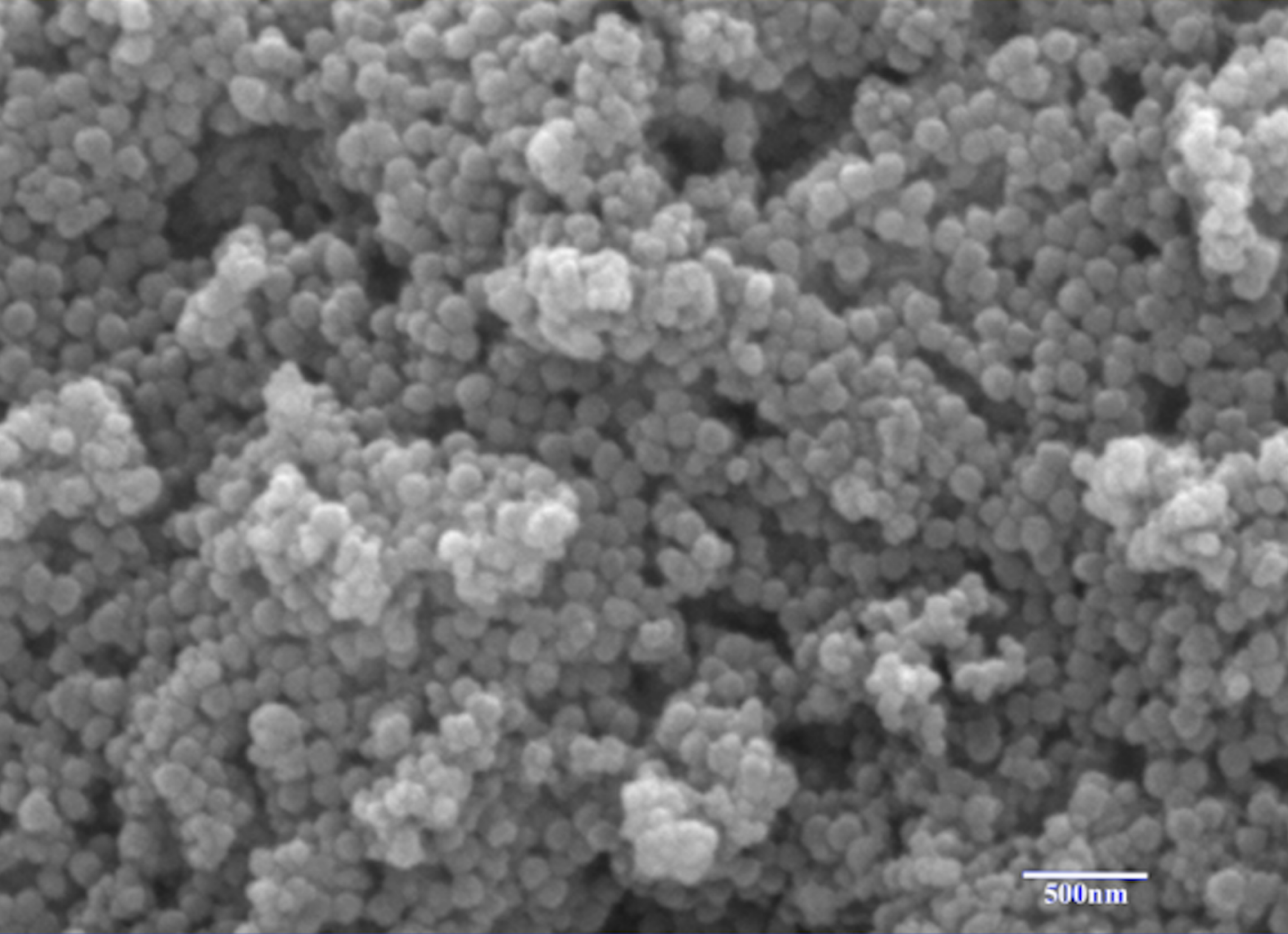
SEM image of SNPs.

Surface morphology and structure of the obtained SNPs were investigated by SEM. As seen in Figure 3, the SNPs are spherical. It was also observed that a low proportion of SNPs were around 200–300 nm, and the majority were around 150 nm. SNPs may provide a larger specific surface area, resulting in a high loading capacity for DOX. Also, this property is an advantage regarding the penetration of SNPs into cells.

Physicochemical features such as size, shape, and surface charge play an extremely important role in the internalization of nanostructures. The uptake of nanoparticles into cells requires two steps. The first is the binding to the cell membrane, and the second is the uptake into the cell [34]. The zeta potential of SNPs was measured as +14.1 mV at pH 7.0 [24]. Nanostructures with this surface charge will repel each other and will not aggregate. The high surface charge of the SNPs can be seen as evidence of suspension stability. In addition, the positively charged nanostructures are preferentially bound to tumors [35–36] and retained longer than negatively charged and/or neutral structures [36]. This phenomenon demonstrates the potential of utilizing these SNPs in biomedicine.

### DOX loading to SNPs

**Figure 4:**
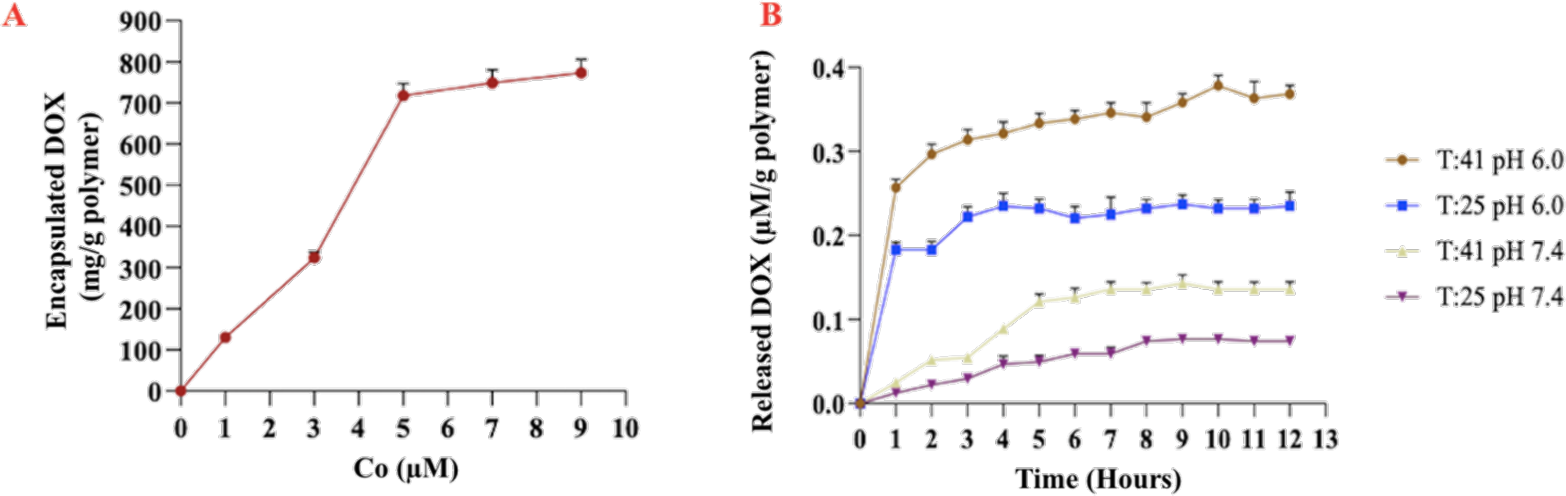
(A) Loading capacity of DOX at different initial concentrations. (B) Time-dependent release of DOX under different conditions.

One of the most important points when designing a DD material is its drug-carrying capacity because a structure with a low carrying capacity may not provide the desired effect. DOX loading of SNPs was conducted as previously mentioned in section “Preparation of DOX-SNPs”. The DOX-loading capacity of smart nanopolymers was investigated in the concentration range of 1–9 µM (5 mg SNPs in 5 mL 0.05 M phosphate buffer/methanol 1/1, v/v, pH 6.0). The loading of DOX happened very swiftly at low concentrations because of the high surface area of the obtained nanostructures (Figure 4A). The DOX loading capacity of nanopolymers was 773 mg/g nanopolymer. Besides, a high loading of 79.08% of DOX molecules was obtained because of fast filling of the adsorption sites. In conclusion, DOX loading onto the SNPs was accomplished successfully. In previous studies conducted by our group on the carrying capacity of nanostructures, we obtained comparable results [20,24,37].

### Encapsulation and release studies of DOX encapsulated SNPs

The encapsulation and release behaviors of SNPs are extremely important for both efficient delivery and release. To evaluate the release properties of the synthesized SNPs, a PBS solution with a DOX concentration of 5 µM at pH 7.0 was prepared. The methodology for incorporating DOX into SNPs is explained in detail in section “DOX loading to SNPs “. Then, the solutions with DOX-loaded SNPs were analyzed by UV–vis spectrophotometry at 495 nm wavelength with pure PBS serving as a control to calculate the amount of unencapsulated DOX. PBS buffer was used to remove both unencapsulated and unreacted DOX. The encapsulation efficiency was determined using Equation 1:


[1] $$\begin{array}{l} \%\text{Loading efficiency}=\\ \frac{\text{Total encapsulated DOX }-\text{ Non encapsulated DOX}}{\text{Total encapsulated DOX}}\;\times \;100\%. \end{array}$$

As expected, DOX was successfully encapsulated in the SNPs. The combination of temperature-sensitive pNIPA and pH-sensitive VIm was investigated at two temperatures (25 and 41 °C) and two pH values (pH 6.0 and 7.4). The total release of DOX was highest at 41 °C and pH 7.4 (Figure 4B). The temperature and pH sensitivity eliminates the need for other external stimuli. The time-dependent DOX release experiments conducted here under different conditions demonstrated that the dual response of the SNPs may improve the DD performance compared to the response to a single stimulus.

### Cytotoxity of DOX-SNPs

**Figure 5:**
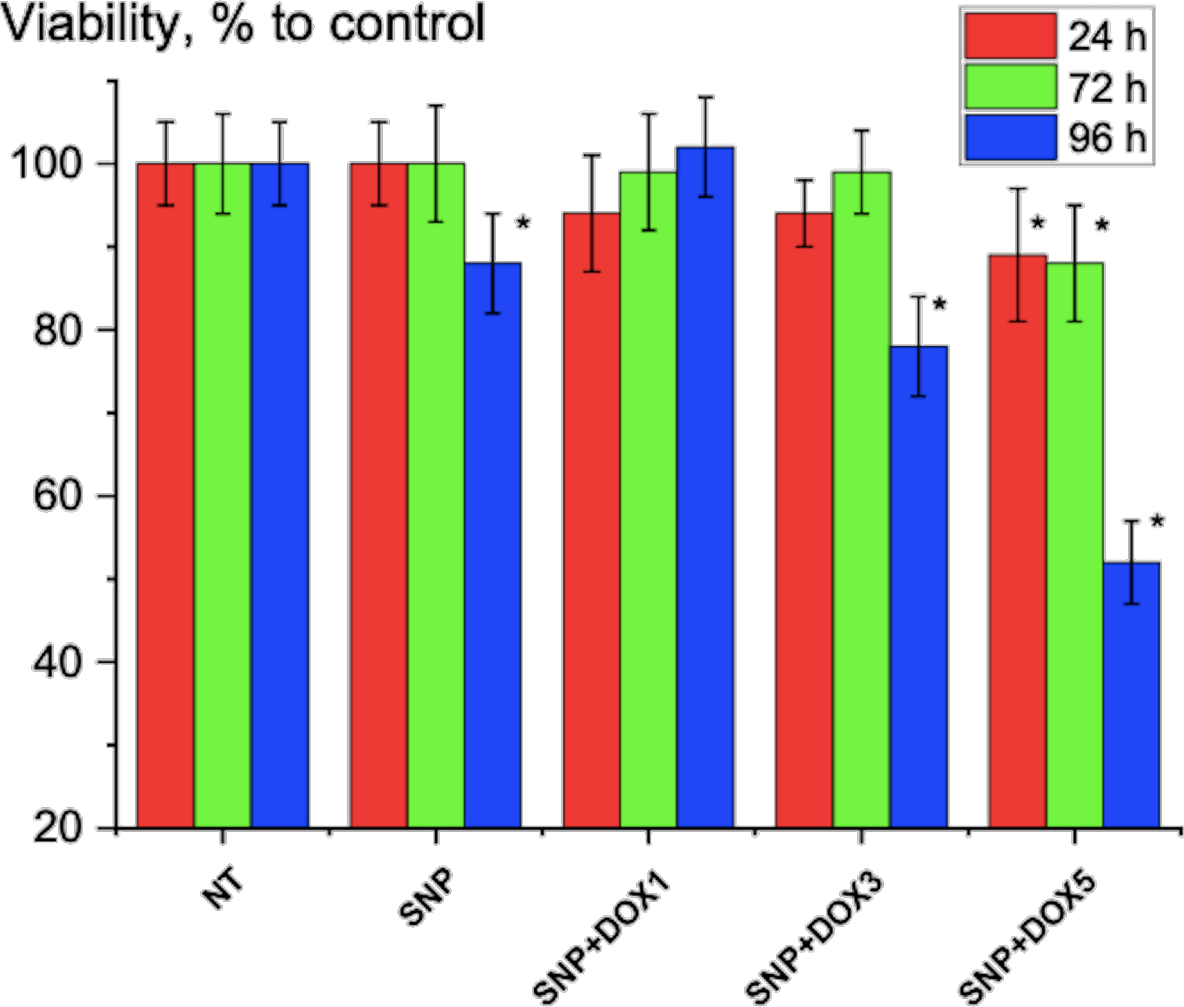
Cell viability of the HeLa cell line treated with DOX-SNPs and pure SNPs incubated for 24, 72 and 96 h in DMEM at 37 °C (* – *p* < 0.05 to control; NT – non treated cells see section “Cytotoxicity test” for details).

DOX-SNPs were prepared as mentioned above, and their effect on the cancer cell line HeLa was studied at incubation times of 24, 48, 72, and 96 h at 37 °C (Figure 5, data after 48 h were similar to those after 24 and 72 h and are not presented here). The release of DOX inside cells was slow and continuous. The effect of released DOX after 24–72 h was quite small, that is, approx. 10% decrease in viability of the cells. Taking into account the data on DOX release at *T* = 41 °C and pH 6.0 (Figure 4B), we can suppose that this point corresponds to a concentration of free DOX in the solution of 2 µmol/L (5 µM/L of DOX × 0.4) as it is known that the basic drugs doxorubicin and mitoxantrone are concentrated in acidic endosomes of cells with pH approximately 5.5 [38].

**Figure 6:**
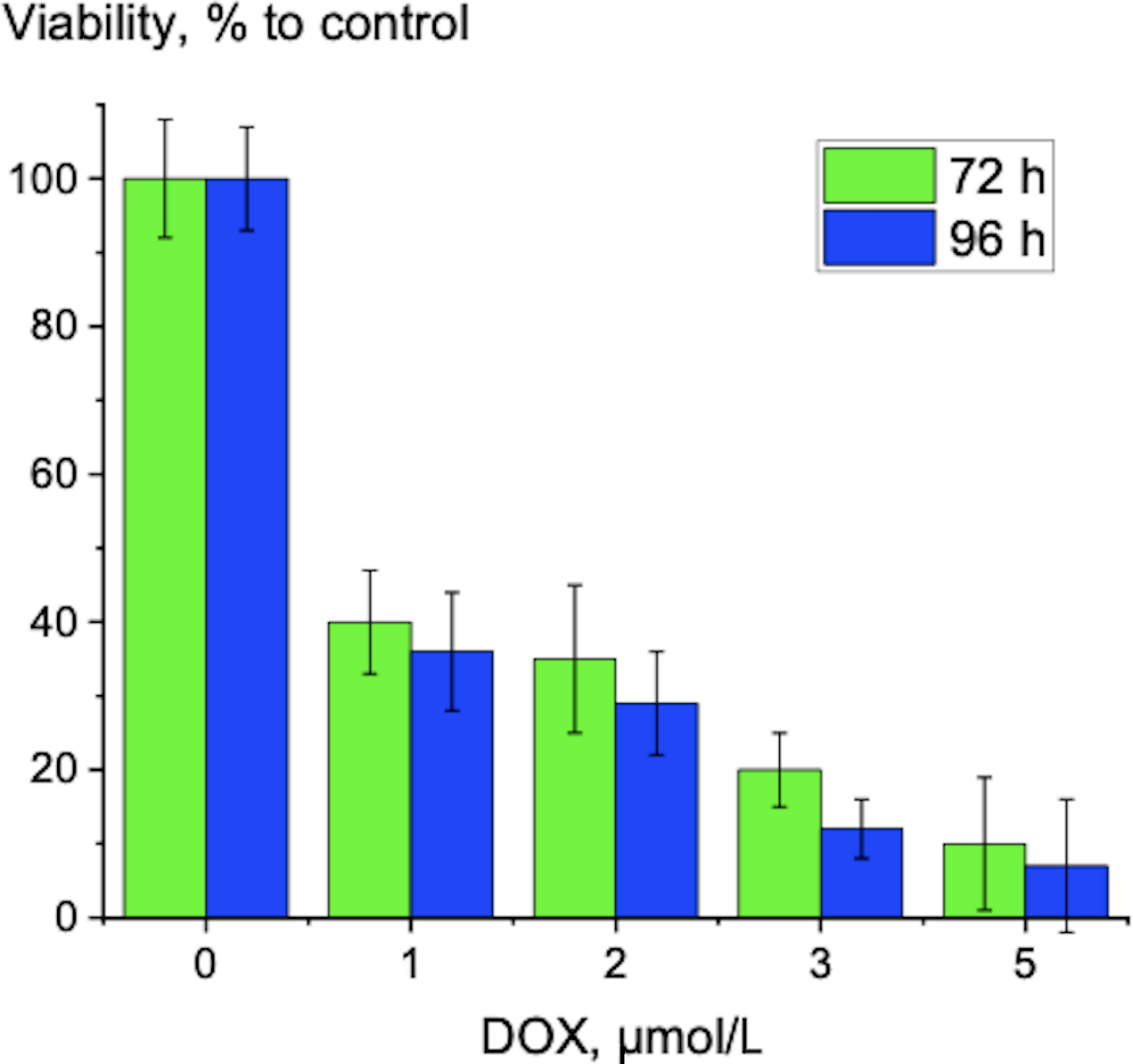
Cell viability of HeLa cells treated with DOX for 72 and 96 h in DMEM at 37 °C.

Incubation for 96 h led to a decrease of viability of HeLa cells at 5 µmol/L loaded DOX (i.e., approx. 2 µmol/L released DOX) to 52 ± 7%. This is close to the effect of pure DOX on cells under the same conditions (Figure 6). It means that DOX is continuously released from SNPs during incubation with HeLa cells, and this process is prolonged in time. The slow release at pH 7.4 and the fast release at pH 6.0, that is, under acidic conditions typical for cancer environments, is an advantage of our complex. If we compare the nanocomplex with Doxil, we can find similar features. A mechanistic model of DOX release from Doxil showed that DOX is released up to 19 h [39]. In our case we observed a release reaching a plateau after 12 h (Figure 4B). Also, it was found that in cancer cells in mice, ammonium/ammonia levels in tumor lesions are in the millimolar range, higher than in the blood plasma. Using tumor cells in culture, the authors showed that Doxil in the presence of ammonia killed tumor cells on a level similar to that of free doxorubicin. Doxil without ammonia and ammonia without Doxil had a very poor cytotoxicity [40]. We obtained similar results. After 96 h of incubation, the effect of DOX-SNPs on HeLa cells at 5 µmol/L (i.e., 52% ± 7%) was comparable to the effect of at 2 µmol/L pure DOX on HeLa cells (i.e., 37% ± 7%). Also, as in our experiments, the data on DOX release at pH 7.4, 6.0, and 5.0 from polyelectrolyte layers indicated no release of DOX at pH 7.4 and high DOX release at pH 6.0 and 5.0. The growth of human hepatoma cells (HepG2) was inhibited at pH 5.0 in a medium with DOX–polyelectrolyte multilayers [41]. Regarding the possibility of using DOX-SNP complexes in vivo we can refer to the behavior of the similar PEG-PLGA-DOX polymersome complex [42]. It was found that PEG-PLGA-DOX polymersomes accumulated in tumor lesions, and after 48 h of treatment, their presence in the liver was lower than that of a Doxil mimic. DOX polymersomes showed better efficiency than the Doxil mimic against tumor after one injection at lower doses. Both formulations induced the similar changes in body and in blood of mice as follows from histological and heamatological tests. The authors concluded that DOX polymersomes have less side effects than Doxil because of the biodegradability and release features of the complex [42].

## Conclusion

Specifically designed nanomedicines can deliver various agents simultaneously to a desired site of action, resulting in more effective release of active ingredients. The design of the release conditions of the drug, as well as the encapsulation, are critical because of the low solubility of cancer drugs. Smart nanopolymeric platforms exhibit good in vitro or in vivo DR performance at various drug loading levels. The results here show that the response of polymeric nanoplatforms to dual/multiple stimuli can further improve DD performance compared to nanoplatform systems that are responsive to only one stimulus. Temperature- and pH-sensitive nanopolymers were synthesized and characterized by FTIR, SEM, and zeta potential measurements. The DOX loading capacity of the nanopolymers was found to be sufficient. Encapsulation and release studies were conducted, and it turned out that the release from the dual stimuli-responsive system was higher than that from single stimulus-responsive systems. The cytotoxicity of SNP-DOX complexes against the cancer cell line Hela at different DOX concentrations and incubation times showed a prolonged DOX release and a good anticancer effect. The effect was similar to that observed in a commercial liposomal formulation of doxorubicin (Doxil) as well as to that of other polymeric formulations of DOX. The data shows that smart nanopolymers can be used to create new types of doxorubicin-based drugs.

## Data Availability

The data that supports the findings of this study is available from the corresponding author upon reasonable request.
